# Nurses' and Families' Experiences of a Feasibility Trial Comparing Single‐Use and Multiple‐Use Endotracheal Suction Catheters in Mechanically Ventilated Patients: A Qualitative Study

**DOI:** 10.1111/nicc.70624

**Published:** 2026-08-13

**Authors:** Mohamed H. Eid, Kevin Hambridge, Patricia Schofield, Jos M. Latour

**Affiliations:** ^1^ School of Nursing and Midwifery, Faculty of Health University of Plymouth Plymouth UK; ^2^ Critical Care and Emergency Nursing Department, Faculty of Nursing Mansoura University Mansoura Egypt; ^3^ Faculty of Nursing Mansoura National University Mansoura Egypt; ^4^ The Curtin School of Nursing Curtin University Perth Australia; ^5^ Department of Nursing Zhongshan Hospital, Fudan University Shanghai China

**Keywords:** airway management, chlorhexidine, intensive care units, interview, qualitative research

## Abstract

**Background:**

In resource‐limited Intensive Care Units (ICUs), reusing endotracheal suctioning catheters is often standard practice due to supply constraints or staffing pressures. Randomised controlled trials (RCTs) have explored the impact of these practices on patient outcomes. However, less is known about the experiences of nurses and family members involved in RCTs testing endotracheal suctioning interventions.

**Aim:**

To explore the experiences of ICU nurses and family members participating in a feasibility RCT comparing single‐use versus multiple‐use endotracheal suction catheters flushed with chlorhexidine or normal saline in a resource‐limited ICU setting.

**Study Design:**

A qualitative study was embedded within a feasibility RCT. Semi‐structured interviews were conducted with 13 ICU nurses and seven family members at a university hospital in Egypt. Data were audio‐recorded, transcribed verbatim, and analysed using Thematic Analysis supported by NVivo software.

**Findings:**

Five themes were identified: (1) Navigating resource limitations in daily practice, (2) Building knowledge and confidence in intervention delivery, (3) Balancing infection control with feasibility, (4) Families navigating through emotional and cultural uncertainties, and (5) Family perceptions of trial participation and consent. Nurses valued structured training, which enhanced adherence and confidence, but expressed concerns about budget and supply constraints. Families appreciated clear explanations of interventions and valued the potential to improve patient safety, though some reported anxiety around unfamiliar procedures.

**Conclusions:**

Both nurses and family considered the interventions and trial procedures acceptable and feasible within the ICU context, although long‐term implementation may require addressing resources and training needs for ICU nurses.

**Relevance to Clinical Practice:**

Understanding the experiences of delivering interventions by nurses and the involvement of family in clinical trials is essential for designing RCTs testing novel nursing interventions. These insights can inform future large‐scale RCTs testing endotracheal suction catheters interventions in mechanically ventilated patients and ensure trial successes.

**Trial Registration:**

ClinicalTrials.gov, identifier NCT06207513

## Impact Statements

- What is known about this topic
  ○ Reuse of single‐use endotracheal suction catheters is common in resource‐limited ICUs due to supply constraints, cost, and staffing pressures, but its impact on patient outcomes is not fully established
  ○ Chlorhexidine flushing may reduce microbial colonisation and the risk of ventilator‐associated pneumonia, although evidence on nurses' experiences and practical challenges with this intervention is limited.
  ○ Less is known about the experiences of nurses and family members involved in trials testing endotracheal suction catheter interventions in mechanically ventilated patients.
- What this paper adds
  ○ Nurses perceive chlorhexidine flushing as beneficial for secretion clearance and infection control, but implementation is constrained by resource shortages, time pressures, and policy limitations.
  ○ Families' participation in ICU trials is influenced by trust in healthcare professionals, cultural/religious beliefs, and communication, highlighting the importance of ethically and culturally sensitive consent processes.
  ○ Embedding qualitative research in feasibility RCTs provides valuable insights into practical, organisational, and relational factors affecting intervention acceptability and adherence in real‐world ICU settings.

## 1 Introduction

Ventilator‐associated pneumonia (VAP) remains one of the most considerable complications in mechanically ventilated patients, particularly in intensive care units (ICUs) within resource‐limited countries, where shortages of medical supplies, financial constraints, and limited procurement systems may necessitate adaptations to standard care practices, including the reuse of devices intended for single use [1, 2]. Patients receiving mechanical ventilation are prone to secretion accumulation due to sedation and impaired natural airway clearance, which increases the risk of pulmonary infections [3, 4]. Endotracheal suctioning is a standard of respiratory care in these patients, facilitating secretion removal, optimising oxygenation and ventilation, and preventing complications such as pneumonia, atelectasis, and tube obstruction [5, 6, 7].

Endotracheal suctioning is performed using open or closed endotracheal systems; open systems require temporary ventilator disconnection to insert a single‐use catheter, while closed systems allow continuous ventilation within a sterile sheath [8, 9]. Evidence shows no significant difference in VAP incidence or mortality between the two systems [10, 11]. However, the optimal frequency of catheter use is unclear, especially in resource‐limited ICUs where single‐use catheters are often reused during the duration of a nursing shift due to cost and logistics [12, 13, 14]. Chlorhexidine is a broad‐spectrum antiseptic that has a prolonged effect of up to 48 h and may reduce microbial colonisation when used to flush endotracheal suction circuits, potentially lowering VAP risk [15, 16]. A quasi‐experimental study reported reduced VAP incidence when chlorhexidine flushing was used with multiple‐use catheters in resource‐limited ICUs [2]. Despite these potential benefits, nurses' experiences, practical challenges, and perceptions of reusing single‐use catheters with chlorhexidine flushing remain poorly understood [17, 18].

Qualitative research can provide valuable insights into nurses' experiences, helping to inform both clinical practice and the design of larger trials. Understanding nurses' experiences is especially important in feasibility clinical trials, as frontline staff play a central role in ensuring adherence to study protocols, maintaining patient safety, and integrating interventions into routine care [19, 20]. Exploring these experiences allows identification of practical barriers, facilitators, and contextual factors that influence implementation in real‐world ICU settings. Equally, gaining insight into families' experiences is essential, as their perspectives contribute significantly to achieving these aims. Therefore, this qualitative study was embedded within a feasibility RCT to generate deeper understanding of these experiences [21]. Although recommendations and policies exist regarding the use and replacement of closed suction catheters, the present feasibility RCT focused exclusively on open endotracheal suction catheters, comparing single‐use catheters with multiple‐use catheters flushed with chlorhexidine. The qualitative component explored nurses' and family members' experiences and perceptions of implementing this practice.

## 2 Aim and Objectives

The aim of this embedded qualitative study was to explore the experiences of ICU nurses and family members regarding participation in a feasibility RCT testing chlorhexidine suction circuit flushing with single‐use suction catheters in mechanically ventilated patients.

The objectives of this study were to examine: (i) nurses' experiences of the feasibility, acceptability, and practical challenges of delivering the intervention, and (ii) families' experiences of the consent process, their decision‐making regarding trial participation, and their perceptions of their relative's involvement in the study.

## 3 Methods

### 3.1 Research Design and Setting

A qualitative approach was adopted using face‐to‐face interviews with nurses and family members. The study was conducted in three ICUs within a university hospital in Egypt. The ICUs included surgical, neurological and trauma units, each consisting of 10 beds and providing care to mechanically ventilated patients. Collectively, the three ICUs admit approximately 650 patients per year. The study was prospectively registered at ClinicalTrials.gov with the number NCT06207513. The study protocol has been published previously [18]. The quantitative data of the feasibility RCT has been published previously [21]. In this paper we present the qualitative data utilising the Consolidated Criteria for Reporting Qualitative Research (COREQ) checklist [22] to report the study (Supporting Information S1).

### 3.2 Research Team and Reflexivity

All interviews were conducted face‐to‐face by the first author (MHE), a male registered nurse with over a decade of ICU experience in Egypt and the UK, who was responsible for participant recruitment, interviewing, audio‐recording, transcription, and translation. At the time of the study, he was a PhD candidate under the supervision of two professors of clinical nursing (JML and PS) and one lecturer (KH). The first author (MHE) received training in qualitative research, and the supervisory support informed the interview process and reflexive practice throughout the study. No prior personal relationship existed between the interviewer and participants (ICU nurses or family members); participants were informed in advance about the interviewer's role, study aims, and reasons for conducting the research, and the interviewer's background was disclosed for transparency. Field notes and a reflexive diary were maintained during data collection and analysis to document potential biases, assumptions, and evolving interpretations.

### 3.3 Population and Recruitment

Purposeful sampling was used to recruit the participants to ensure inclusion of individuals with direct and relevant experience of the intervention. Two groups were recruited: (1) ICU nurses who were directly involved in delivering suction procedures as part of the trial, and (2) first‐degree family members of patients enrolled in the feasibility RCT who had provided consent for participation. Temporary staff (agency or locum nurses) and staff who were not involved in the trial were excluded to ensure familiarity with the study procedures. Eligible participants were approached in person by the researcher (MHE) within the ICU setting, and written informed consent was obtained from participants prior to conducting the interviews.

According to the study protocol, the planned sample size was 12–16 ICU nurses and 12–16 family members. Recruitment, data collection, and initial data analysis continued concurrently, and recruiting of participants proceeded until adequate variation in participants' experiences was achieved and data saturation was reached, defined as the point at which no new codes and subthemes emerged from the data [23].

### 3.4 Data Collection

Interviews were conducted in a private room within the hospital to ensure confidentiality and to minimise interruptions. Only the participant and the interviewer were present during each interview, and no non‐participants, such as family members or colleagues, attended. Separate semi‐structured interview guides were developed for the two participant groups (ICU nurses and family members). The guides were informed by the trial protocol, relevant literature, and methodological guidance on embedding qualitative research within feasibility studies (Supporting Information S2). The interview guides were piloted with one ICU nurse and one family member who met the eligibility criteria but were not included in the final analysis. Minor refinements were made to improve clarity and sequencing of questions.

Interviews were audio‐recorded with participants' informed consent. Reflexive field notes and a research diary were maintained throughout data collection to document contextual details, methodological decisions, and researcher reflections. Interview transcripts were prepared verbatim in Arabic and subsequently translated into English to facilitate analysis with the research team members.

### 3.5 Data Analysis

Data were analysed using an inductive thematic analysis following the six phases described by Braun and Clarke [23]. This process involved: Phase (1) familiarisation with the data through repeated reading of transcripts; Phase (2) generation of initial codes by the first author (MHE); Phase (3) searching for patterns by collating related codes into potential subthemes and preliminary themes by two authors (MHE and KH); Phase (4) reviewing subthemes to ensure coherence within themes and clear distinctions between themes by three authors (MHE, KH, and JML); Phase (5) defining and naming themes to capture their core meaning by all authors (MHE, KH, JML, PS); and Phase (6) producing the final analytic account. Interview transcripts were imported into NVivo software, version 14 (QSR International, 2023) [24] to support data management, systematic coding, and the organisation of themes throughout the analytic process.

Analytic rigour was enhanced through reflexive journaling and peer debriefing, with themes developed collaboratively (MHE, and KH), then reviewed and refined in discussion with the wider research team members [23]. Formal member checking was not undertaken, as participants were not asked to review transcripts or preliminary coding.

### 3.6 Ethical Considerations

Ethical approval was obtained from two ethics committees: the Faculty of Health Research Ethics and Integrity Committee at the University of Plymouth, United Kingdom (Reference: 4333, Dated 14.12.2023), and the Research Ethics Committee at the Faculty of Nursing, Mansoura University, Egypt (Reference: 411, Dated 27.11.2023). Participants were verbally informed about the study aims, procedures, voluntary nature of participation, and issues related to confidentiality. After this, participant information sheets were provided which had been reviewed and approved by both research ethics committees.

## 4 Findings

Twenty‐seven eligible participants were approached comprising 14 nurses and 13 family members, of whom a total of 20 participants were included in the study, comprising 13 ICU nurses and 7 family members. Interviews lasted between 25 and 50 min. The nurses were bedside ICU nurses with varying lengths of ICU experience (between 2 and 17 years), representing both junior and more experienced staff, and included both male (*n* = 4) and female (*n* = 9) participants. Family members were next‐of‐kin of mechanically ventilated patients, including spouses (*n* = 3) and first‐degree relatives (*n* = 4), and represented both male (*n* = 6) and female (*n* = 1) participants. These participant characteristics provide contextual information to support interpretation of the qualitative findings.

The findings are presented in two separate sections according to participant group: first, the themes generated from the nurses' interviews, followed by those generated from the family members' interviews. Thematic analysis identified three themes from the nurses' interviews (Table 1) and two themes from the family members' interviews (Table 2). The analysis initially generated several preliminary themes and subthemes. Through iterative review and discussion among the research team, overlapping concepts were refined and consolidated into three themes relating to nurses' experiences and two themes relating to family members' experiences. The findings are supported by illustrative participant quotations, identified by participant codes, such as nurse N002 and family member F001, to preserve confidentiality.

**TABLE 1:** Themes and subthemes of nurses' experiences.

Theme	Subthemes	Example quotations
Navigating resource limitations in daily practice	Resource limitations and adaptive reuse	We face this situation many times; we don't have supplies, so we re‐use what is available. (N010) In some days, there's no catheters available, so we re‐use them. (N011) Single‐use catheters are better, but sometimes we can't afford them. (N007) We follow what the management says because we can't change the policy ourselves, even if it's unsafe. (N012) Even if chlorhexidine is better, we cannot use it unless it's approved by hospital policy. (N002).
	Time – pressure dynamics	Sometimes we have many ventilated patients and only two nurses; we do our best but it's exhausting. (N006) When there's emergency or shortage of staff, suctioning becomes very rushed. (N009)
Building knowledge and confidence in intervention delivery	Procedure clarity	We need more training or workshops to know the correct method. (N006) If there was a clear guideline about chlorhexidine, I think everyone would follow it. (N013) Sometimes we feel unsure because different nurses teach different methods. (N012)
	Professional proficiency	When we are trained, we feel safe and know exactly what to do. (N008) Once I practiced the technique a few times, I felt much more confident using chlorhexidine. (N012) When we understand why the intervention is done, we perform it more correctly and consistently. (N013) Good training makes us feel safe, especially with new procedures we didn't learn in school. (N011)
Balancing infection control with feasibility	Benefits and challenges of chlorhexidine use	Chlorhexidine makes me feel the line and tube are cleaner, and it prevents bad smell. (N004) I saw less thick secretions after using it regularly. (N010) It helps reduce the smell and the sticky secretions, but sometimes we run out of it. (N012)
	Observed patient signs	I noticed patients had fewer fevers when chlorhexidine was used. (N008) When secretions were thick, we cleaned with saline if no chlorhexidine available. (N013) With reused catheters the secretions become thicker, and patients need more suctioning. (N003)

**TABLE 2:** Themes and subthemes of family members' experiences.

Theme	Subthemes	Example quotations
Families navigating through emotional and cultural uncertainties	Emotional and cultural constraints	At first I didn't want them to try anything new, I was afraid. (F001) Everything in the ICU is frightening, and when they ask you to sign papers, the fear becomes bigger. (F003) We believe that medicine is part of God's will; if it helps others, it's good. (F005) I refused at first because I didn't want anyone to try anything new on my wife while she is unconscious. (F004)
	Supportive communication	I wanted to know if there were any side effects before signing. (F003) When they explained again slowly, I understood better and felt more comfortable. (F002) I kept asking if this would hurt her, and they answered me kindly every time. (F005)
Family perceptions of trial participation and consent	Informed consent	They asked me before including my father, and I agreed because they explained everything. (F005) They said it's safe and used before, so I didn't worry. (F004) I asked if there is danger, and they said they monitor patients closely—that was enough for me. (F006) I didn't know everything, but I know they won't harm him. (F003)
	Motivations and acceptability	I hope what they learned from my husband's case helps others. (F007) My father always loved helping others, so I felt this is something he would approve. (F006) Maybe this research will improve care for other people—that made me accept. (F005)

### 4.1 Nurses' Experiences

#### 4.1.1 Theme 1: Navigating Resource Limitations in Daily Practice

Nurses described persistent shortages of single‐use catheters and chlorhexidine, which directly shaped their suctioning practices (Table 1). Many reported routinely adapting to these limitations. One nurse explained:

- *Sometimes we don't have enough single‐use catheters, so we re‐use them for the same patient*. [N004].

Another nurse added: Reusing feels unsafe, but we have no other option. [N008].

These accounts reflect how resource limitation led nurses to rely on improvised solutions that conflicted with recommended standards. Time pressure further compounded these constraints. Staff described caring for numerous ventilated patients with limited personnel, which created rushed and sometimes suboptimal suctioning. As one participant shared:

- *When we are only two nurses with many ventilated patients, suctioning is rushed and not always ideal*. [N011].

These workload pressures often restricted the ability to consistently apply optimal infection‐control and prevention techniques.

#### 4.1.2 Theme 2: Building Knowledge and Confidence in Intervention Delivery

A second theme described variability in nurses' understanding of suctioning procedures and their reliance on informal learning (Table 1). Unclear mechanical ventilation and endotracheal suction protocols left many uncertain about correct technique. For example, one nurse noted:

- *The policy about saline flushing is not clear; we just follow what our seniors do*. [N002].

Another reflected on the absence of structured training, stating:

- *We were never formally trained on suctioning policy; we just copy what others do in the unit*. [N011].

As nurses gained experience and training of the endotracheal suction procedures described in the feasibility RCT protocol, their confidence increased. A participant explained:

- *After I understood the procedure, I felt more confident to perform it safely*. [N003].

Others emphasised that clarity and hands‐on practice enabled them to deliver the intervention more consistently and with greater assurance.

#### 4.1.3 Theme 3: Balancing Infection Control With Feasibility

Nurses recognised benefits associated with chlorhexidine use but noted challenges to sustaining it within current resource limitations (Table 1). Several described visible improvements in cleanliness and secretion quality. One nurse stated:

- *Chlorhexidine feels cleaner than saline; you can tell the circuit is cleaner afterwards*. [N013].

However, limited availability often restricted its use:

- *We would like to keep using it, but it's expensive and not always available, so we can't rely on it*. [N011].

Nurses also described perceived clinical differences in patient outcomes. For example, one participant observed:

- *Patients in the chlorhexidine group seemed more stable with less thick secretions by day six*. [N012].

These impressions illustrate how frontline staff interpreted patient responses in relation to the intervention, while simultaneously working within a system of fluctuating resources.

### 4.2 Families' Experiences

#### 4.2.1 Theme 1: Families Navigating Emotional and Cultural Uncertainties

Families described considerable anxiety when learning that their relative might be included in a clinical study (Table 2). Critical illness and unfamiliar procedures intensified distress. One family member recalled:

- *I was scared when they told me my husband is part of a study*. [F002].

Cultural and religious values also influenced decisions. A participant shared:

- *I asked the Sheikh to make sure this was not forbidden before I agreed*. [F006].

These accounts reflect how emotional burden and cultural beliefs shaped early responses to research involvement. Supportive communication played an essential role in easing these concerns. Families valued clear explanations delivered with patience. As one relative described:

- *The nurse explained in simple Arabic and that made me comfortable*. [F004].

Another added:

- *They told me they would call me immediately if anything wrong happened—this assurance made me accept*. [F007].

Such communication fostered trust and reduced apprehension.

#### 4.2.2 Theme 2: Family Perceptions of Trial Participation and Consent

Families often relied on trust in healthcare professionals during the consent process (Table 2). Some acknowledged limited understanding yet still agreed based on this trust. One participant stated:

- *I signed quickly but didn't fully understand; I trusted the doctor*. [F001].

Others emphasised reassurance, such as:

- *If there were any side effects, they would stop it*. [F002].

Altruism emerged as a strong motivator for participation. Several families expressed satisfaction knowing their involvement could help others. A family member shared:

- *If this research helps other patients, I am happy to take part*. [F003].

Another commented:

- *What matters is that others might benefit from our experience*. [F003].

These sentiments illustrate how personal values and trust contributed to a positive view of participation.

## 5 Discussion

This embedded qualitative study aimed to explore the experiences of ICU nurses and family members taking part in a feasibility RCT testing the use of chlorhexidine flushing compared with single‐use versus multiple‐use endotracheal suction catheters in mechanically ventilated patients. By interviewing 13 nurses and 7 family members, the study provided new insights into both the professional and familial dimensions of care within a resource‐limited ICU setting. Five themes were identified. Nurses valued structured training, which enhanced adherence and confidence, but expressed concerns about budget and supply constraints. Families appreciated clear explanations of interventions and valued the potential to improve patient safety, though some reported anxiety around unfamiliar procedures.

### 5.1 Nurses' Experiences

Our findings regarding resource limitations and reuse of single‐use suction catheters echo concerns from recent empirical reviews [25]. A scoping review mapping the implications of reusing single‐use endotracheal suction catheters in mechanically ventilated patients found limited and inconsistent evidence, highlighting that reuse may increase the risk of respiratory infections, but the data remains inconclusive [17]. This lack of strong evidence perhaps explains why in many low‐ and middle‐income ICU settings, reuse of endotracheal suction catheters during a nursing shift remains common practice, a reality captured in our nurses' narratives.

However, our findings also showed that nurses perceive practical benefits when using chlorhexidine flushing in reused suction systems. This perception aligns with a recent quasi‐experimental study conducted in Egypt that demonstrated that flushing multiple‐use suction catheters and circuits with 0.2% chlorhexidine gluconate significantly reduced the incidence of ventilator‐associated pneumonia (VAP) in mechanically ventilated ICU patients [2]. This clinical data provides empirical support for the benefits that nurses in our study perceived in terms of improved secretion clearance and reduced infection risks.

Local hospital and unit policies were another important issue. Nurses often felt restricted by hospital rules, even when the rules or protocols were not updated with the current evidence [2, 17, 18]. This shows a common problem where policies and practice are not fully aligned, and bureaucracy slows down the use of better, evidence‐based methods. Nurses were generally positive about using chlorhexidine, which is supported by research for its antimicrobial effect [26, 27, 28]. However, opinions on single‐use endotracheal suction catheters were mixed. This highlights a common challenge in infection prevention: the safest options are often hard to use in hospitals with limited resources. Similar patterns have been seen in global health studies [29, 30], where effective interventions are sometimes abandoned because they are difficult to sustain.

Our qualitative data adds an important dimension: it illustrates how real‐world constraints including supply shortages, time pressure, and limitations in training influence whether potentially beneficial protocols (like chlorhexidine flushing in our feasibility RCT study protocol) can be applied consistently or effectively. In resource‐limited ICUs such as those included in our feasibility RCT, the gap between best‐evidence and real‐world practice may be shaped as much by institutional constraints as by the knowledge or attitudes of the ICU staff.

### 5.2 Family Members' Experiences

From the families' point of view, ICU research participation is emotionally and culturally complex. Relatives often face stress and uncertainty when asked to consent for a critically ill family member [31]. Our findings show that initial reactions frequently include fear and hesitation, reflecting both the critical nature of the patient's condition and unfamiliarity with medical interventions. Similar challenges have been reported in previous studies of proxy decision‐making in critical care research, where emotional distress and uncertainty can affect relatives' ability to process information and make informed decisions [32, 33]. Supportive and clear communication from nurses was crucial in alleviating anxiety and facilitating informed decision‐making. Families valued explanations delivered patiently and in culturally familiar terms, which fostered trust and comfort during the consent process.

Decision‐making was strongly influenced by trust in healthcare professionals. Many relatives agreed to participation based on confidence in the competence and integrity of the medical team rather than complete understanding of the study procedures or potential risks. This finding is consistent with previous research demonstrating that family members commonly rely on trust in clinicians when making decisions regarding research participation for critically ill relatives [31, 32]. While trust may facilitate recruitment and engagement, it also highlights the importance of ensuring that consent discussions clearly distinguish research participation from routine clinical care and provide opportunities for ongoing clarification and discussion [34]. Families in our study reported that reassurance about monitoring, immediate reporting of complications, and prior experience with similar interventions helped them feel safer in providing consent.

The importance of communication identified in our findings is supported by growing evidence that family members benefit from structured decision‐support and shared decision‐making approaches in critical care settings [33, 35]. Previous research suggests that interventions designed to improve communication and support surrogate decision‐makers can enhance understanding, facilitate participation in decision‐making, and improve the overall experience of families during periods of critical illness [33, 35]. Our findings reinforce the need to view informed consent as an ongoing process rather than a single event, particularly in ICU settings where emotional distress may affect information recall and comprehension. International ethical guidance similarly conceptualises consent as an ongoing and revisable process rather than a one‐off or irrevocable authorisation, requiring consent to be revisited when study procedures, circumstances, or relevant information change and participants' continued willingness to participate to be confirmed [36, 37]. This approach is particularly important in critical care, where patients' decision‐making capacity and surrogate decision‐makers' understanding may evolve, necessitating opportunities to repeat information, ask questions, and renew or withdraw consent [37, 38].

Cultural and religious considerations played a significant role in families' acceptance of participation. Several relatives consulted religious authorities or reflected on beliefs about divine will before agreeing, demonstrating the importance of aligning consent discussions with culturally meaningful frameworks [39]. Participation was also motivated by altruism, with relatives expressing satisfaction that their involvement could contribute to the care and well‐being of future patients. This underscores that, beyond comprehension of risks and procedures, personal values and beliefs are integral to decision‐making in ICU research contexts.

Finally, families' perceptions highlight practical implications for trial design and ethics. Consistent with recommendations from critical care and research ethics literature, consent processes should extend beyond the provision of written information to include iterative discussions, culturally sensitive communication, and reassurance regarding patient safety [32, 33, 35]. Families in our study valued opportunities to ask questions, receive ongoing explanations, and understand how participation might benefit future patients. These findings suggest that incorporating family perspectives into trial design and participant‐facing materials may improve the acceptability of research interventions, strengthen informed consent processes, and enhance recruitment and retention in future ICU trials.

## 6 Limitations

This study has several limitations. Firstly, it was carried out in only one ICU in Egypt. This means the results may not apply to other hospitals or countries where resources, policies, or cultures are different. Secondly, although the number of participants was suitable for a qualitative study, the sample was still small, and other nurses or families may have different experiences. Additionally, all interviews were conducted in Arabic and then translated into English. Care was taken to keep the original meaning as accurate as possible; however, some feelings, expressions, or cultural meanings may have changed during the translation. The first author conducted all interviews, which may have created some bias, even though reflexive notes and discussions with other researchers were used to reduce this. Finally, because the study was part of a feasibility trial, some nurses or family members may have answered in a way they thought was expected, which could affect honesty in some responses.

## 7 Implications for Practice, Policy, and Research

The dual experiences of nurses and family members yield several implications:

1. For clinical practice: Nurses are motivated to adopt safer practices but require systemic support. Training alone is insufficient unless accompanied by resource provision and policy reform. Incorporating chlorhexidine flushing as a feasible alternative to single‐use catheters may represent a pragmatic strategy in resource‐limited ICUs, provided supply is consistent and staff are adequately trained.
2. For institutional and health policy: The study underscores a review and most likely an update of endotracheal suction and mechanical ventilation protocols to align with evidence‐based practice. Health systems must bridge the gap between research findings and bedside implementation, ensuring policies are responsive to evolving evidence
3. For informed consent and ethics: Families' reliance on trust and cultural values in decision‐making highlights the need for ethically robust consent processes that move beyond the provision of information to fostering genuine understanding. This may involve iterative consent conversations, use of visual aids, or involvement of cultural mediators to ensure that consent reflects both comprehension and voluntariness.
4. For research: Our study demonstrates the value of incorporating both professional and lay‐person experiences when evaluating new interventions in trials. Future studies should consider co‐design approaches, where nurses and families participate in shaping protocols, thereby enhancing feasibility, acceptability, and ethical robustness.

## 8 Conclusion

This study highlights the complex dynamics surrounding the adoption of chlorhexidine flushing and single‐use catheters in mechanically ventilated patients in a feasibility RCT. Nurses emphasised structural and policy barriers yet expressed motivation for change and recognition of potential patient benefits. Family members highlighted the emotional, informational, and cultural factors shaping consent in ICU research. Together, these findings illustrate that optimising endotracheal suctioning practices in a trial and clinical practice requires not only technical and policy reform but also ethically sensitive, culturally attuned engagement with families.

By examining both professional and familial experiences, this study enhances understanding of how endotracheal suctioning interventions are experienced in a real‐world clinical trial. The embedded qualitative design highlights contextual, organisational, and relational factors influencing the implementation, acceptability, and adherence of interventions in our feasibility RCT. This approach aligns with qualitative process evaluations in large multi‐centre clinical trials, explaining how and why interventions succeed or fail. Such insights are especially valuable in trials conducted in resource‐constrained ICUs, where workload, policies, and family trust shape the feasibility and ethical procedures. Providing the findings of qualitative data to complement quantitative outcomes in a feasibility RCT can guide future trial design and successful implementation.

## Author Contributions

M.H.E. and J.M.L. contributed to the design of the study. M.H.E. performed the data collection and conducted the analysis. K.H., P.S., and J.M.L. contributed to the interpretation and synthesis of the data. The initial draft of the manuscript was prepared by M.H.E. K.H., P.S., and J.M.L. provided critical feedback and revisions to the manuscript. All authors reviewed the final version of the manuscript and approved it for submission.

## Funding

The researcher [Mohamed H. Eid] is funded by a full scholarship [MM71/22] from the Ministry of Higher Education of the Arab Republic of Egypt.

## Ethics Statement

This study has been reviewed and approved by the following ethics committees: the Faculty of Health Research Ethics and Integrity Committee at the University of Plymouth, United Kingdom (Reference: 4333), and the Research Ethics Committee at the Faculty of Nursing, Mansoura University, Egypt (Reference: 411/2023).

## Consent

The research ethics committees have reviewed and approved the informed consent forms and participant information sheets of this study. All study participants provided informed consent prior to enrolment in accordance with the Declaration of Helsinki. Participation was voluntary and participants could withdraw at any time.

## Conflicts of Interest

The authors declare no conflicts of interest.

## Supporting information


**Data S1:** Consolidated criteria for reporting qualitative studies (COREQ): 32‐item checklist.

**Data S2:** Tool ΙIΙ [Part I]: Nurses semi‐structured interview questions guide.

## Data Availability

The data that support the findings of this study are available on request from the corresponding author. The data are not publicly available due to privacy or ethical restrictions.
