# Supplementary material for: Nurses' and Families' Experiences of a Feasibility Trial Comparing Single‐Use and Multiple‐Use Endotracheal Suction Catheters in Mechanically Ventilated Patients: A Qualitative Study
Source: Nurs Crit Care. 2026 Aug 13;31(5):e70624. doi: 10.1111/nicc.70624 (PMC13470139; doi:10.1111/nicc.70624)
Supplement: Supplementary file 1 [file NICC-31-0-s001.docx]

# Consolidated criteria for reporting qualitative studies (COREQ): 32-item checklist

## Developed from:

Tong A, Sainsbury P, Craig J. Consolidated criteria for reporting qualitative research (COREQ): a 32-item checklist for interviews and focus groups. *International Journal for Quality in Health Care*. 2007. Volume 19, Number 6: pp. 349 – 357

## MANUSCRIPT TITLE:

Nurses' and Families' Experiences of a Feasibility Trial Comparing Single-Use and Multiple-Use Endotracheal Suction Catheters in Mechanically Ventilated Patients: A Qualitative Study

| **No. Item** | **Guide questions/description** | **Reported on Page #** |
| --- | --- | --- |
| **Domain 1: Research team and reflexivity** |  |  |
| *Personal Characteristics* |  |  |
| 1. Inter viewer/facilitator | Which author/s conducted the interview or focus group? | p.7 |
| 2. Credentials | What were the researcher’s credentials? (E.g. PhD, MD) | p.7 |
| 3. Occupation | What was their occupation at the time of the study? | p.7 |
| 4. Gender | Was the researcher male or female? | p.7 |
| 5. Experience and training | What experience or training did the researcher have? | p.7 |
| *Relationship with participants* |  |  |
| 6. Relationship established | *Was a relationship established prior to study commencement?*  . | p.7 |
| 7. Participant knowledge of the interviewer | *What did the participants know about the researcher? (e.g. personal goals, reasons for doing the research).* | p.7 |
| 8. Interviewer characteristics | *What characteristics were reported about the interviewer/facilitator? (e.g. Bias, assumptions, reasons and interests in the research topic)* | p.7 |
| **Domain 2: Study design** |  |  |
| *Theoretical framework* |  |  |
| 9. Methodological orientation and Theory | *What methodological orientation was stated to underpin the study? (e.g. grounded theory, discourse analysis, ethnography, phenomenology, content analysis).* | p.7-8 |

| *Participant selection* |  |  |
| --- | --- | --- |
| 10. Sampling | *How were participants selected? (e.g. purposive, convenience, consecutive, snowball)* | p.7-8 |
| 11. Method of approach | *How were participants approached? (e.g. face-to-face, telephone, mail, email)* | p.8 |
| 12. Sample size | *How many participants were in the study?* | p.8, 10 |
| 13. Non-participation | *How many people refused to participate or dropped out? Reasons?* | p.10 |
| *Setting* |  |  |
| 14. Setting of data collection | *Where was the data collected? (e.g. home, clinic, workplace)* | p.8-9 |
| 15. Presence of non-participants | *Was anyone else present besides the participants and researchers?* | p.8 |
| 16. Description of sample | *What are the important characteristics of the sample? (e.g. demographic data, date)* | p.10 |
| *Data collection* |  |  |
| 17. Interview guide | *Were questions, prompts, guides provided by the authors? Was it pilot tested?* | p.8 |
| 18. Repeat interviews | *Were repeat interviews carried out? If yes, how many?* | N/A |
| 19. Audio/visual recording | *Did the research use audio or visual recording to collect the data?* | p.9-10 |
| 20. Field notes | *Were field notes made during and/or after the interview or focus group?* | p.9 |
| 21. Duration | *What was the duration of the inter views or focus group?* | p.10 |
| 22. Data saturation | Was data saturation discussed? | p.8 |
| 23. Transcripts returned | *Were transcripts returned to participants for comment and/or correction?* | p.9 |
| **Domain 3: analysis and findings** |  |  |
| *Data analysis* |  |  |
| 24. Number of data coders | How many data coders coded the data? | p.9 |

9

| 25. Description of the coding tree | *Did authors provide a description of the coding tree?* | p.10, 26-2 |
| --- | --- | --- |
| 26. Derivation of themes | *Were themes identified in advance or derived from the data?* | p.9 |
| 27. Software | *What software, if applicable, was used to manage the data?* | p.9 |
| 28. Participant checking | *Did participants provide feedback on the findings?* | p.9 |
| *Reporting* |  |  |
| 29. Quotations presented | *Were participant quotations presented to illustrate the themes/findings? Was each*  *quotation identified? (e.g. participant number)* | p.10-14 |
| 30. Data and findings consistent | *Was there consistency between the data presented and the findings?* | p.10-29 |
| 31. Clarity of major themes | *Were major themes clearly presented in the findings?* | p.10-14 |
| 32. Clarity of minor themes | Is there a description of diverse cases or discussion of minor themes? | p.26-29 |
