# Supplementary material for: Nurses' and Families' Experiences of a Feasibility Trial Comparing Single‐Use and Multiple‐Use Endotracheal Suction Catheters in Mechanically Ventilated Patients: A Qualitative Study
Source: Nurs Crit Care. 2026 Aug 13;31(5):e70624. doi: 10.1111/nicc.70624 (PMC13470139; doi:10.1111/nicc.70624)
Supplement: Supplementary file 2 [file NICC-31-0-s002.docx]

**Tool ΙIΙ [Part I]: Nurses Semi-Structured Interview Questions Guide**

| **Questions** | **Prompts** | **2^nd^ Prompts** |
| --- | --- | --- |
| Think back to your standard routine of suctioning using suction catheters multiple times and use normal saline for flushing the suction circuit of patient’s secretions. How do you feel the standard suctioning routine influences patient care, if at all? | If Yes- can you describe if it has a good or a bad effect on your patient? (In what ways did the patients affected)  If no- why not? What would you have liked about the routine care? | *Prompt-including nurses’ beliefs about routine endotracheal suctioning.* |
| Did you have negative, challenging, or troubling thoughts or feelings surrounding the need for changing the routine suctioning care and try new suctioning modalities? | What did you like or dislike about the information regarding trying a new suctioning technique? | How confident did you feel using the new technique? |
| Primary outcomes (3^rd^ day follow up)  Think back to the day three of applying the intervention. What changes, if any, did you observe in your patient by day three? | Were the outcomes good or bad? And was it related to a specific study intervention? | If it was bad, was it related to a specific group or all groups? |
| Primary outcomes (6^th^ day follow up)  Think back to the day six of applying the intervention. Was your patient’s status improved or deteriorated in each study groups? | Were the outcomes good or bad? And was it related to a specific study intervention? | If it was bad, was it related to a specific group or all groups? |
| Can you tell us more about your experience of using the suctioning catheters once? Did it have an impact on your patient? | How would you describe the usefulness or practicality of using the catheter once? | What challenges did you encounter? |
| Can you tell us more about your experience of using the chlorhexidine to flush the suctioning circuit? Did it have an impact on your patient? | How would you describe the usefulness or practicality of using the catheter once? |  |

.

**Tool ΙIΙ [Part II]: Next of Kin Semi-Structured Interview Questions Guide**

| **Questions** | **Prompts** | **2^nd^ Prompts** |
| --- | --- | --- |
| Think back to your relative and asking you to let him participate in our study. How was your feeling? | Were you motivated to allow his participation?  If no, why? |  |
| In terms of the approach clinical lead nurse/PI used to offer you the deferred consent. How did you experience the consent process? Did it feel comfortable and voluntary? | Did you have to sign the consent against your desire? |  |
| How do you feel now about the decision to allow participation? | If yes, can you give a reason?  What did you feel? | What did you feel? |
| Were the information sheet that you provided clear for you? | Did you understand the medical terminology used on it? | Did you find a complicated part? |
| Were the health benefits, risks, compensations clear for you? | Did you understand what should be done in case of side effects happened to your relative? | What parts were clear or unclear for you? |
| Did you feel that the proposed intervention affected your relative’s health outcomes? | If yes, Was it in a good or bad way? | What changes, if any, did you notice in your relative during the study? |
